# Morphological changes in intraepithelial and stromal telocytes in *Cyprinus carpio* in response to salinity stress

**DOI:** 10.1038/s41598-023-43279-4

**Published:** 2023-11-15

**Authors:** Walaa F. A. Emeish, Hanan H. Abd-ElHafeez, Abdullah A. A. Alghamdi, Madeha Ahmed, Mahmoud Osman Khalifa, Ahmed A. El-Mansi, Alaa S. Abou-Elhamd, Mohsen M. Khormi, Khalid Alkashif, Soha A. Soliman

**Affiliations:** 1 Department of Fish Diseases, Faculty of Veterinary Medicine, South Valley University, Qena, 83523 Egypt; 2 Department of Cell and Tissues, Faculty of Veterinary Medicine, Assiut University, Assiut, 71526 Egypt; 3 Department of Biology, Faculty of Science, Albaha University, Albaha, Saudi Arabia; 4 Department of Histology, Faculty of Veterinary Medicine, Sohag University, Sohagt, 82524 Egypt; 5 Department of Anatomy and Embryology, Faculty of Veterinary Medicine, Aswan University, Aswan, Egypt; 6 Department of Molecular Bone Biology, Graduate School of Biomedical Sciences, Nagasaki University, 1-7-1 Sakamoto, Nagasaki, 852-8588 Japan; 7 Biology Department, Faculty of Science, King Khalid University, Abha, Saudi Arabia; 8 Department of Respiratory Therapy, Faculty of Applied Medical Sciences, Jazan University, Jazan, Saudi Arabia; 9 Department of Biology, College of Science, Jazan University, Jazan, Saudi Arabia; 10 Physiology Department, faculty of Medicine, Merit University, Sohage, Egypt; 11 Department of Histology, Faculty of Veterinary Medicine, South Valley University, Qena, 83523 Egypt

**Keywords:** Cell biology, Anatomy

## Abstract

Telocytes establish connections and communicate with various types of cells and structures. Few experimental studies have been performed on telocytes. In this study, we investigated the effect of salinity stress on telocytes in relation to osmoregulatory, immune, and stem cells. After exposing the common carp to 0.2 (control), 6, 10, or 14 ppt salinity, we extracted and fixed gill samples in glutaraldehyde, processed and embedded the samples in resin, and prepared semi-thin and ultrathin sections. Two types of telocytes were identified: intraepithelial and stromal telocytes. Intraepithelial telocytes were found to form part of the cellular lining of the lymphatic space and shed secretory vesicles into this space. Stromal telocytes were observed to shed their secretory vesicles into the secondary circulatory vessels. Both intraepithelial and stromal telocytes were enlarged and exhibited increased secretory activities as salinity increased. They exerted their effects via direct contact and paracrine signaling. The following changes were observed in samples from fish exposed to high salinity levels: chloride cells underwent hypertrophy, and their mitochondria became cigar-shaped; pavement cells were enlarged, and their micro-ridges became thin and elongated; stromal telocytes established contact with stem cells and skeletal myoblasts; skeletal muscle cells underwent hypertrophy; and macrophages and rodlet cells increased in number. In conclusion, our findings indicate that intraepithelial and stromal telocytes respond to salinity stress by activating cellular signaling and that they play major roles in osmoregulation, immunity, and regeneration.

## Introduction

Telocytes are a distinctive type of interstitial cell and have a wide range of biological functions in different tissues and organs. Through their functional diversity, telocytes affect different types of cells and structures^1^. Telocytes have unique morphological characteristics. Telopodes, which are multiple cell prolongations, emerge from the cell body and may be hundreds of microns in length. Telopodes may give rise to dichotomous branches and establish cellular connections to form a complex labyrinthine system. Telopodes are composed of thin segments (podomers) and interval expansions (podoms), which are rich in calcium release units, mitochondria, endoplasmic reticulum, and caveolae.

Telocytes influence other cells either through a paracrine mechanism or by establishing cellular contact. For example, telocytes establish synaptic junctions to connect to immunoreactive cells^2^. Two types of cellular contact are documented for telocytes: homocellular and heterocellular contact. Homocellular contact occurs between two telopodes, telocytes and telopodes, or the cell bodies of two adjacent telocytes. Heterocellular contact occurs between telocytes and stromal cells, either fixed or free cells. The mechanisms responsible for cellular contact and communication involving telocytes include direct apposition of the cell membrane of adjacent telocytes, adherence, and the formation of gap junctions. Gap junctions play a significant role in intercellular signaling pathways^3^. In addition, telocytes possess a secretory function that is also utilized to influence target cells. Telocytes deliver microvesicles to other cells, providing them with macromolecules such as proteins, RNA, and microRNA. Telocytes also shed exosomes, ectosomes, and multivesicular bodies^2,4,5^.

Telocytes are multifunctional cells. They contribute to the generation and transmission of nerve impulses to involuntary muscles^6–9^. They are involved in mechanoreception and may be involved in atrial fibrillation^10^. Telocytes exhibit receptors for excitatory and inhibitory neurotransmitters^11^. They establish contact with immunoreactive cells, such as eosinophils^4^, mast cells, and macrophages^12^. Telocytes play a role in the regeneration of the heart, lungs, skeletal muscle, skin, meninges and choroid plexus, eye, liver, uterus, and urinary system^13^.

Several studies have been conducted to study telocytes in humans and other mammals; however, few studies have been performed in aquatic species. The current study was conducted using the common carp. Carp belong to the *Cyprinidae* family, which is commonly called the minnow (North America) or carp (Eurasia) family. Members of the *Cyprinidae* family are freshwater species and uncommon in brackish water; they are native to North America, Africa, and Eurasia^14^.

Aquatic species regulate ionic exchange to maintain osmotic balance according to environmental salinity. Several organs are involved in osmoregulation, including the gills, intestines, kidneys, skin, and operculum^15^. Marine inhabitants face great challenges in establishing an ionic balance. Therefore, ion transporting cells, known as chloride cells or ionocytes, participate in the elimination of excess ions in seawater fish and contribute to ion absorption in freshwater fish^16^. In marine fish, chloride cells have undergone specific structural modifications to adapt to high salinity levels. Fish exposed to a high salinity environment acquire a high proportion of mitochondrial-rich chloride cells^17^. Previous studies investigated the relationship between salinity and alterations in ionocytes.

Ionocytes utilize several membrane channels when contributing to osmoregulation, including cystic fibrosis transmembrane conductance regulator (CFTR) (an anion channel), Na–K–2Cl cotransporter (NKCC), and Na^+^/K^+^-ATPase (a sodium–potassium pump). CFTR is a membrane protein located on the apical surface of many types of epithelial cells. It is a cyclic AMP-dependent chloride channel, a bicarbonate channel, and a modulator of other ion channels^18^. NKCC is a membrane transport protein involved in the active transport of sodium, potassium, and chloride ions across the cell membrane^19^. Na^+^/K^+^-ATPase is an electrogenic transmembrane enzyme located predominantly on the basolateral surface of chloride cells and actively transports chloride rather than sodium across the plasma membrane^20^. In addition, pavement cells cover most of the filament and lamellar epithelial surfaces. They are also considered to be ion transporting cells, with a cell membrane rich in hydrogen ion channels^21,22^.

In this study, we focused on the communicating cells, the telocytes, which influence the large population of stromal, muscle, and epithelial cells. The aim of this study was to investigate morphological alterations in telocytes subjected to salinity stress and their effects on different cell types with special reference to the osmoregulatory and immune and stem cells in the common carp.

## Results

This study was conducted to evaluate the acclimation of the common carp to hypertonic conditions, specifically the responses of telocytes and related effector cells (e.g., immune, chloride, and stem cells) in gill filaments and arches, using semi-thin and ultrathin sections.

Fish in the control and 6 ppt groups exhibited normal morphology and behavior, had no noticeable signs of stress, and had no mortality. However, fish in the 10 ppt and 14 ppt groups exhibited a marked reduction in swimming speed and were easily caught.

**Figure 1:**
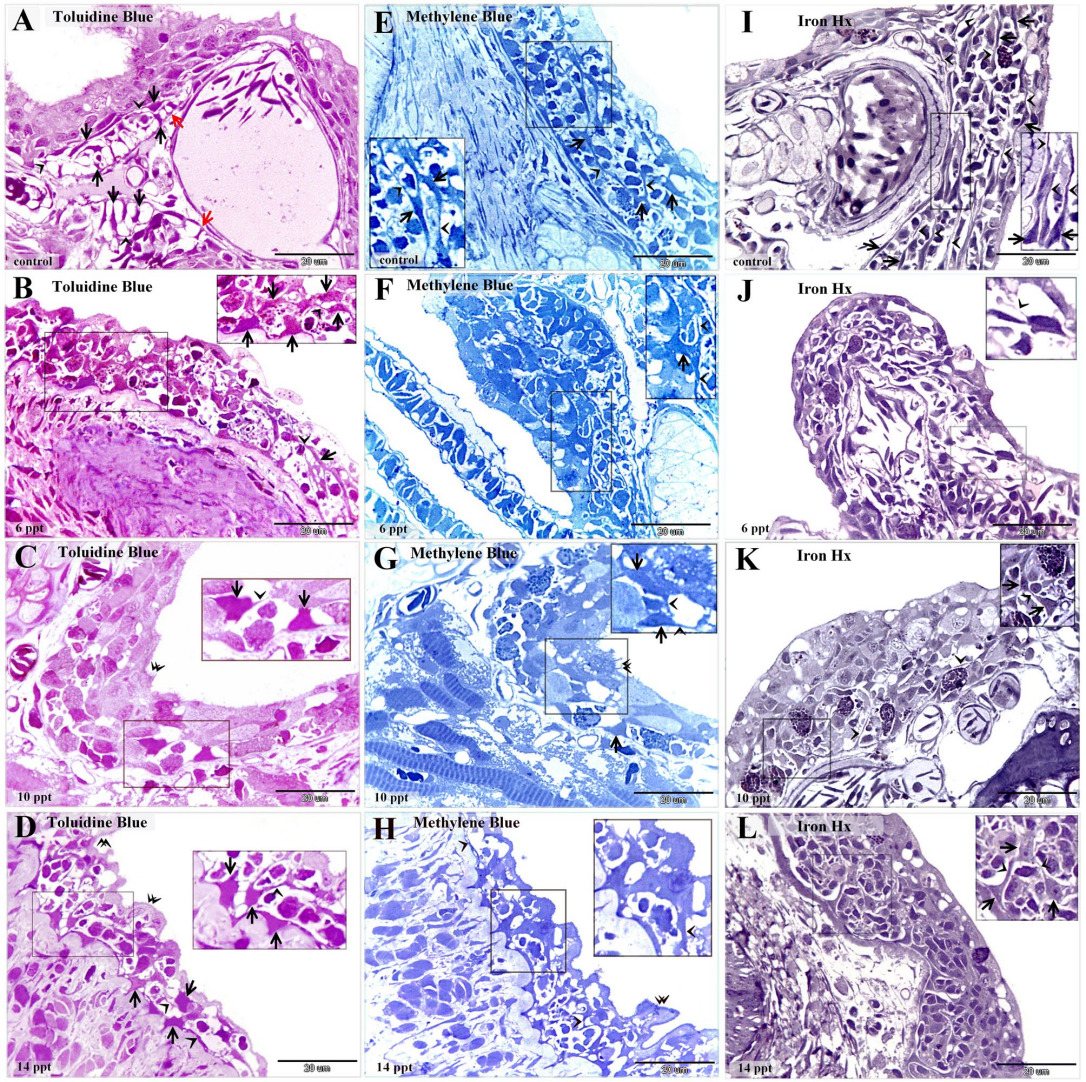
Histopathological changes in intraepithelial telocytes responding to salinity. Semi-thin sections prepared from gill arches and filaments were stained with toluidine blue (**A**–**D**), methylene blue (**E**–**H**), or Heidenhain’s Iron-Hx (**I**–**L**). (**A**, **E**, **I**) Intraepithelial telocytes in the control samples. These cells had small cell bodies (arrows) and prominent telopodes (arrowheads). The red arrows indicate the basal lamina. (**B**, **F**, **J**) Intraepithelial telocytes from fish exposed to 6 ppt salinity. The cell body was enlarged and satellite-shaped (arrows). Note the telopodes (arrowheads). (**C**, **G**, **K**) Cells underwent hypertrophy and became large satellite-shaped cells (arrows) in samples from fish exposed to 10 ppt salinity. Note the telopodes (arrowheads). (**D**, **H**, **L**) The cell bodies (arrows) of intraepithelial telocytes from fish exposed to 14 ppt salinity increased in size. Note the telopodes (arrowheads).

**Figure 2:**
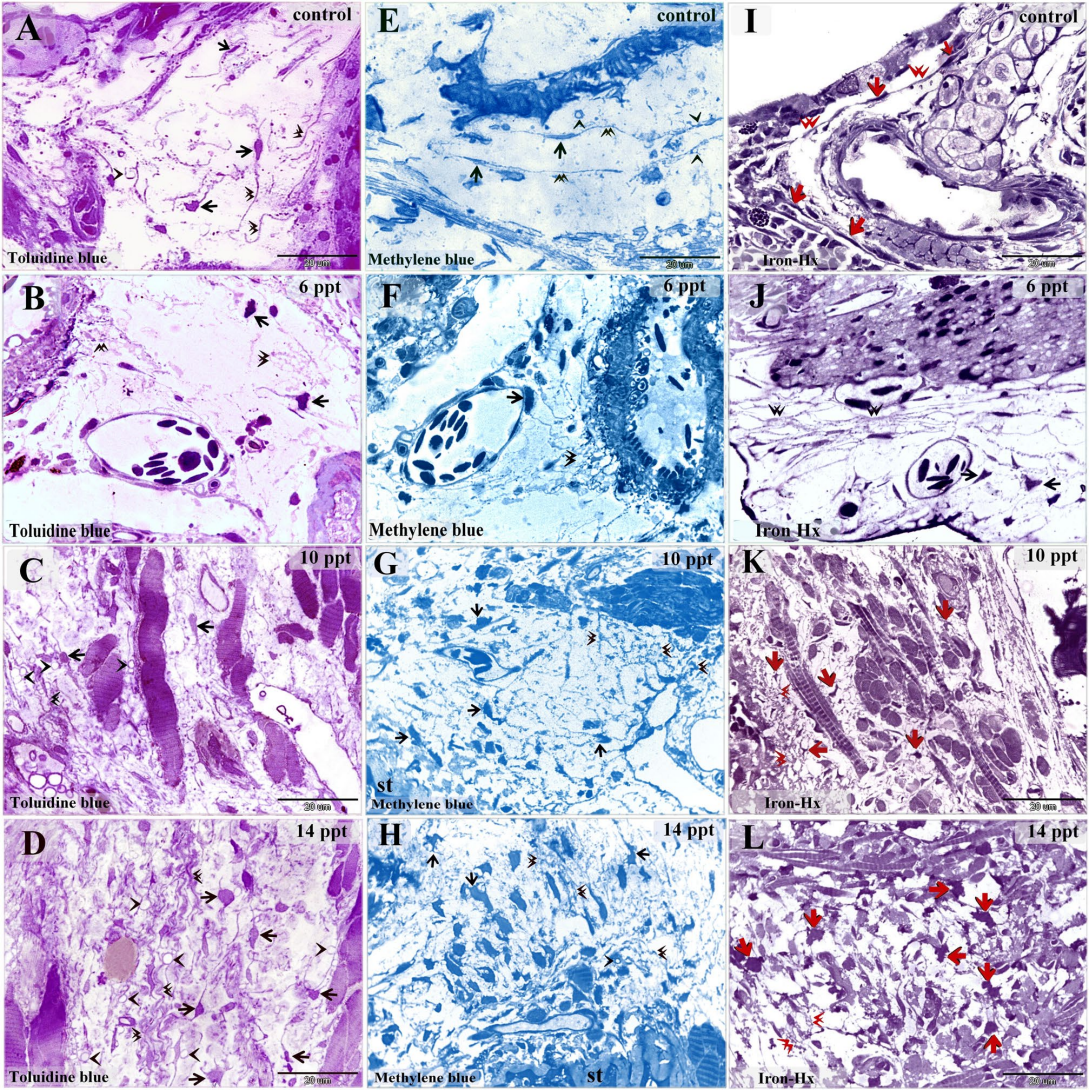
Histopathological changes in stromal telocytes responding to salinity. Semi-thin sections prepared from gill arches and filaments were stained with toluidine blue (**A**–**D**), methylene blue (E–H), or Heidenhain’s Iron-Hx (**I**–**L**). (**A**, **E**, **I**) Stromal telocytes in the control samples. These cells had small cell bodies (arrows) and long telopodes (double arrowheads). Note the secretory vesicles (arrowheads). (**B**, **F**, **J**) Some stromal telocytes had an enlarged cell body (arrows) after exposure to 6 ppt salinity. Note that the telopodes formed a network (double arrowheads). (**C**, **G**, **K**) Stromal telocytes (arrows) had extensive networks of telopodes (double arrowheads) after exposure to 10 ppt salinity. Note the secretory vesicles (arrowheads). (**D**, **H**, **L**) Stromal telocytes (arrows) and telopodes (double arrowheads). Note the secretory vesicles (arrowheads) and stratum compactum (st).

Examination of the semi-thin sections revealed that intraepithelial telocytes from control fish had a small cell body and well-defined telopodes (Fig. 1A,E,I). These cells gradually increased in size with the salinity level. Satellite-shaped intraepithelial telocytes were found in samples from fish exposed to 6 ppt salinity (Fig. 1B,F,J), and large satellite-shaped cells with multiple telopodes were found in samples from fish exposed to 10 ppt and 14 ppt salinity (Fig. 1C,D,G,H,K,L). The control samples contained small stromal telocytes that had a spindle-shaped cell body and fine telopodes (Fig. 2A,E,I). Samples from fish exposed to 6 ppt salinity exhibited some enlarged stromal telocytes with telopodes forming a network (Fig. 2B,F,J). Samples from fish exposed to 10 and 14 ppt salinity were found to contain telopodes forming extensive networks and large, easily recognized secretory vesicles (Fig. 2C,D,G,H,K,L).

**Figure 3:**
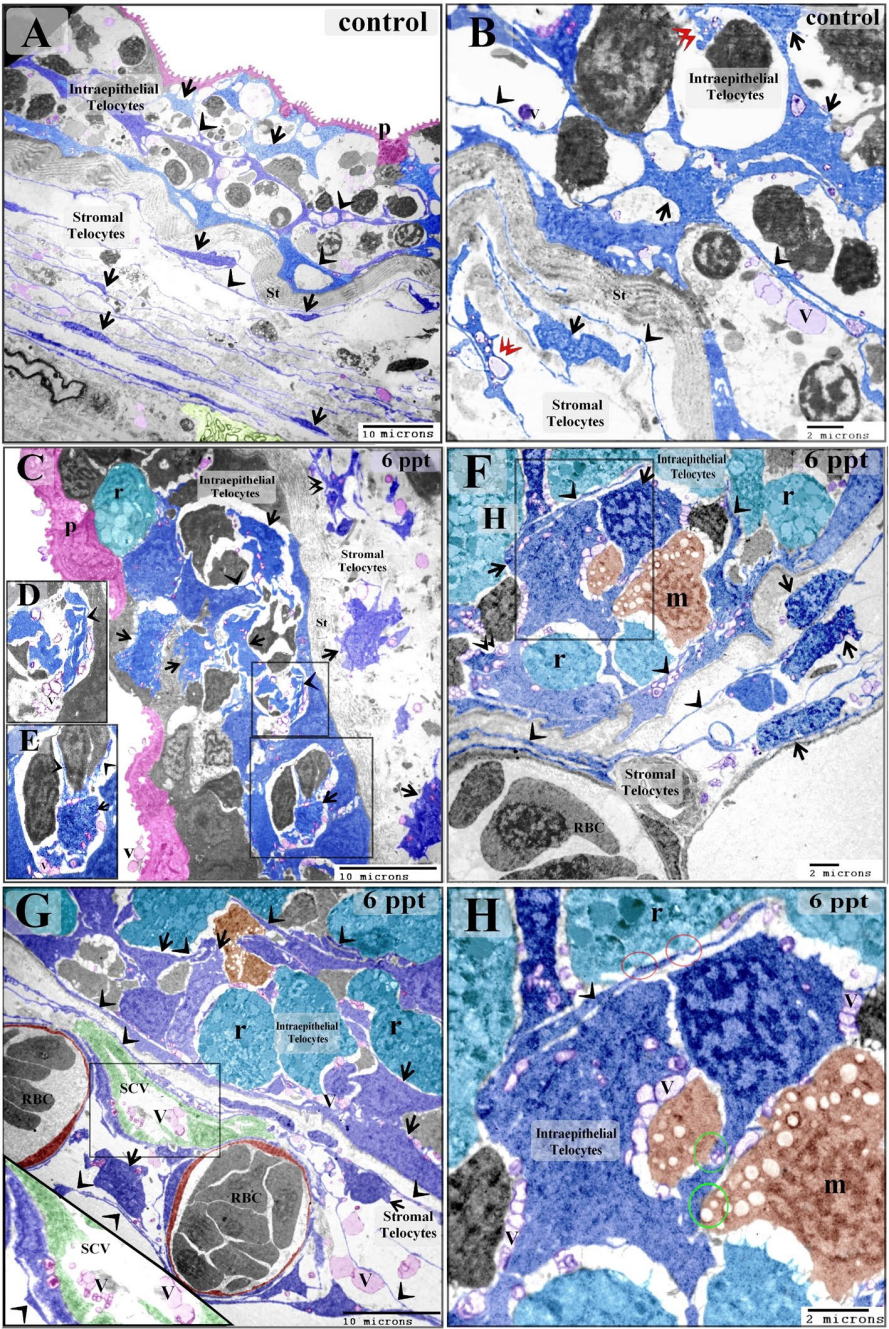
Effect of low level (6 ppt) salinity on intraepithelial telocytes. Colored ultrathin sections prepared from gill arches (**A**–**F**, **H**) and filaments (**G**) of control (**A**, **B**) and 6 ppt-treated samples (**C**–**H**). (**A**, **B**) The intraepithelial telocytes were small, spindle- or satellite-shaped (arrows), and had thin telopodes (arrowheads). They were located on the basal lamina, which is directly opposed to the stratum compactum (st). They were organized in a labyrinthine network, which comprised the wall of the epithelial lymphatic space. Their secretory vesicles (V) were released into the lymphatic space. Note the podoms (double arrowheads) and pavement cells (P). The stromal telocytes had longer and thinner telopodes. (**C**–**H**) Enlarged intraepithelial and stromal telocytes (arrows) with thickened telopodes (arrowheads). The intraepithelial telocytes released their secretory vesicles into the lymphatic space, and the stromal telocytes released their vesicles into the secondary circulatory vessels (SCV) or lymphatic vessels. Note the stratum compactum (st), pavement cells (p), branchial blood vessels containing red blood cells (RBC), and rodlet cells (r) and lysosome-rich macrophages (m) in the lymphatic space. Red circles indicate points of contact between telopodes and rodlet cells. Green circles indicate points of contact between telocytes and the macrophages in the branchial epithelium.

Telocytes were identified by TEM for the first time in the epithelium of the gill arches. In control samples, telocytes were found to form part of the cellular lining of the intraepithelial lymphatic space, an area to which immunoreactive cells migrate. The observed intraepithelial telocytes were small, situated on the basement membrane, and spindle- or satellite-shaped, and their telopodes were thin and formed a labyrinthine network separating between the compartments of the lymphatic space. Their telopodes extended among epithelial and immune cells; hence, they could establish contact with epithelial cells. The secretory vesicles of the telopodes were excreted into the intraepithelial lymphatic space (Fig. 3A,B). In samples from fish exposed to 6 ppt salinity, intraepithelial telocytes that had undergone hypertrophy were observed, as well as thickened telopodes (Fig. 3C–H).

**Figure 4:**
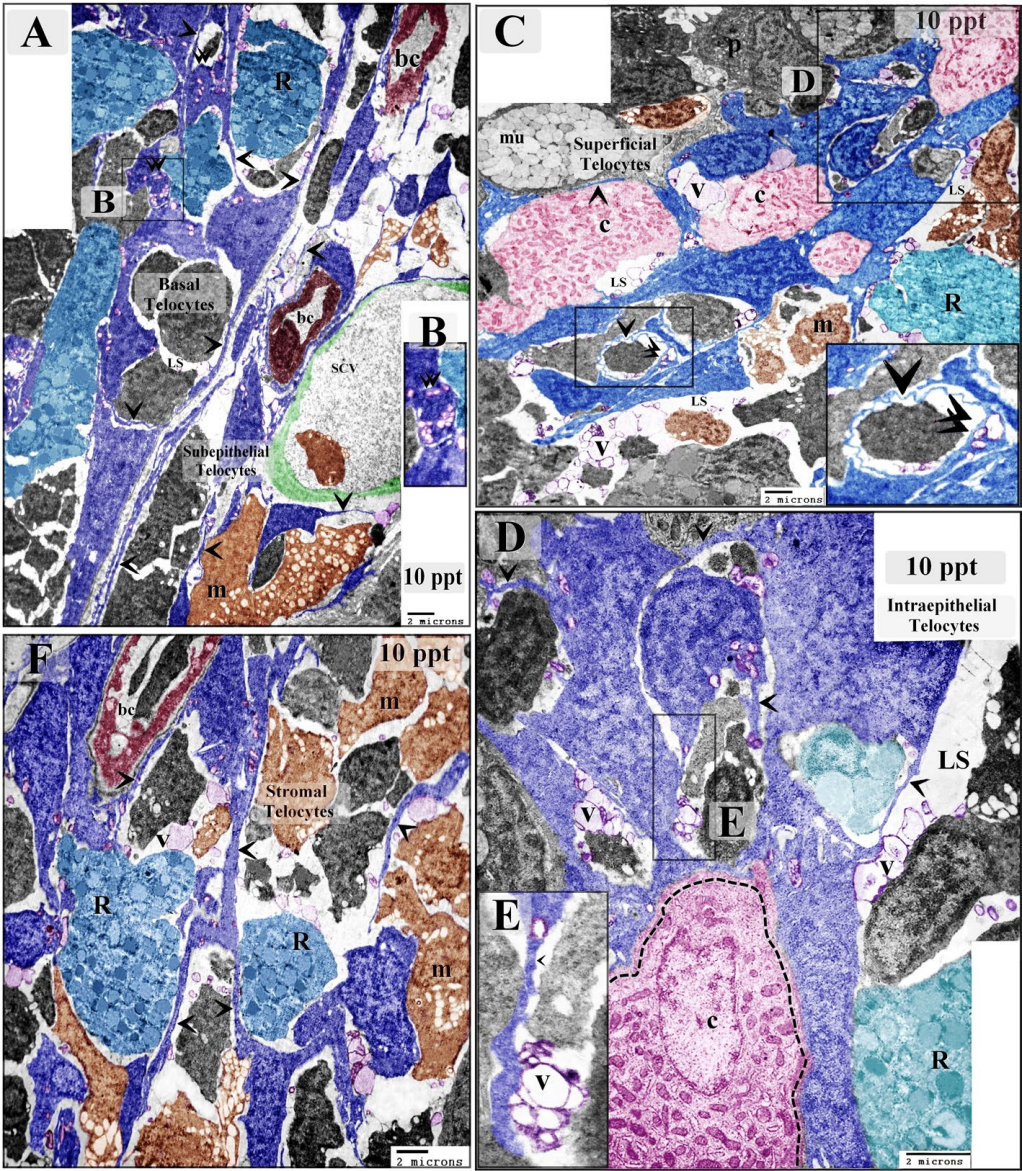
Effect of 10 ppt salinity on intraepithelial telocytes. Colored ultrathin sections prepared from gill arches of fish exposed to 10 ppt salinity. The most prominent feature observed in these samples was the size increase of both the intraepithelial and stromal telocytes. (**A**) Basal telocytes (telocytes in the basal layer of the branchial epithelium) were found to establish a communication network in the lymphatic space (LS), where rodlet cells (R) migrated. Note the telopodes (arrowheads) and podoms (double arrowheads). Subepithelial telocytes connected to blood capillaries (bc), secondary circulatory vessels (SCV), and lysosome-rich macrophages (m). Both basal intraepithelial and subepithelial telocytes underwent hypertrophy. (**B**) Podom under high magnification. (**C**) Superficial intraepithelial telocytes established contact with different types of epithelial cells and formed the lymphatic space (LS) where they shed secretory vesicles (V). Note the telopodes (arrowheads), podoms (double arrowheads), chloride cells (c), rodlet cells (R), pavement cell (P), mucous cell (mu), and macrophages (m). (**D**, **E**) Examples of planar contacts between intraepithelial telocytes and a chloride cell (dashed line). Telocytes shed numerous secretory vesicles (V) into the lymphatic space (LS). Note the telopodes (arrowheads). (**F**) Stromal telocytes connected to different types of stromal cells, including rodlet cells (R) and macrophages (m). Note the telopodes (arrowheads) and blood capillary (bc).

**Figure 5:**
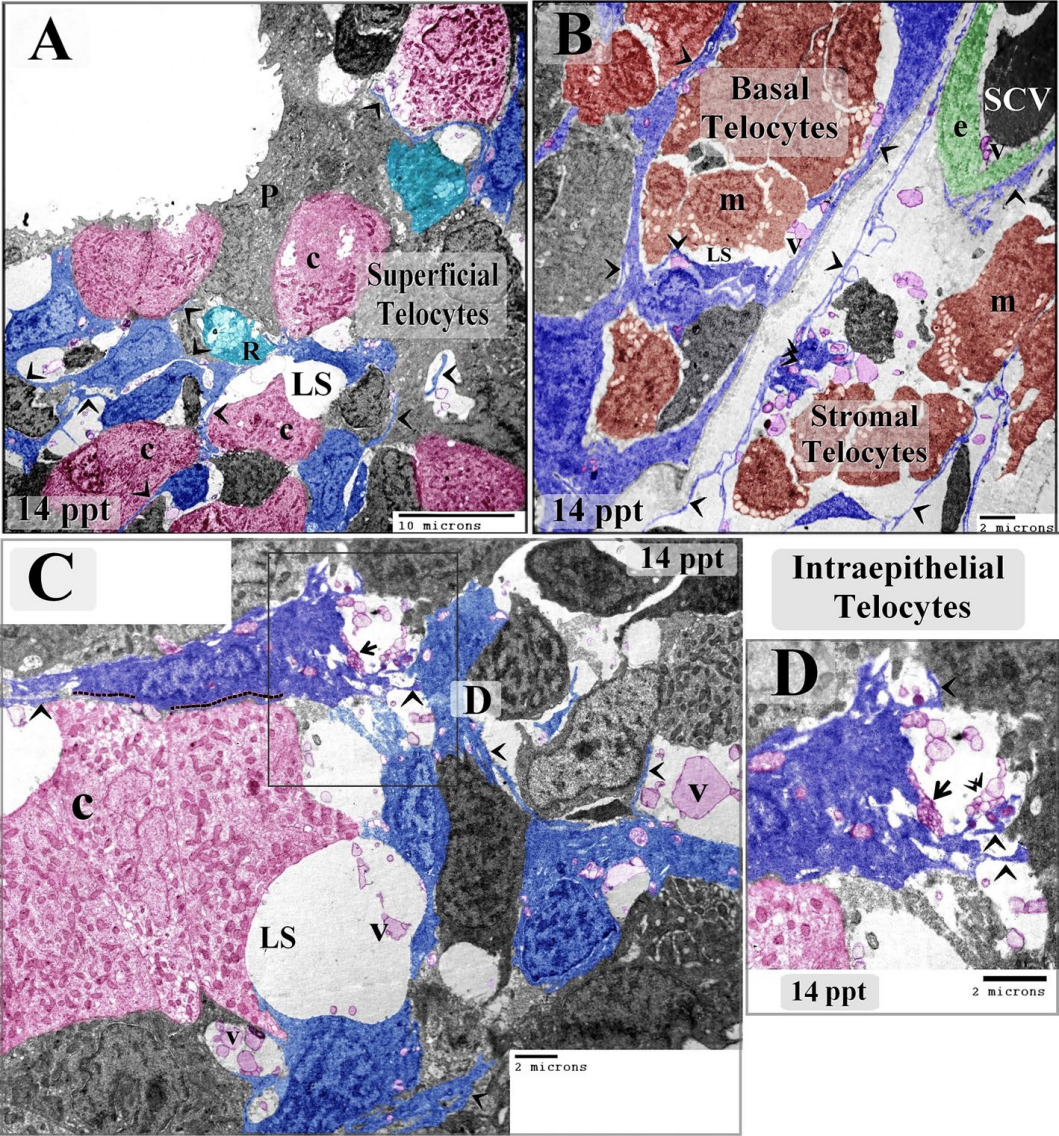
Effect of high salinity (14 ppt) on intraepithelial telocytes. Colored ultrathin sections prepared from gill arches of fish exposed to 14 ppt salinity. (**A**) Superficial intraepithelial telocytes formed a labyrinthine network among epithelial cells and established the lymphatic space (LS). Note the telopodes (arrowheads), chloride cells (C), rodlet cells (R), and pavement cell (P) with short microvilli. (**B**) Basal telocytes formed a network that enclosed the lymphatic space (LS). A massive number of macrophages (M) can be seen in the lymphatic space. Stromal telocytes established contacts with secondary circulatory vessels (SCV) and stromal macrophages (m). Note the telopodes (arrowheads) and podom (double arrowhead). (**C**, **D**) Intraepithelial telocytes formed a network of epithelial cells and constructed the wall of the lymphatic space (LS). An example of an intraepithelial telocyte forming a planar contact is shown (dashed line). The secretory vesicles, multivesicular body (arrow), and exosomes (double arrowhead) of the intraepithelial telocytes were shed into the lymphatic space. Note the telopodes (arrowheads).

Samples from fish exposed to 10 ppt salinity showed hypertrophic intraepithelial telocytes with enlarged podoms and high secretory activity. The intraepithelial telocytes had also established planar contact with chloride cells (Fig. 4A–E). Samples from fish exposed to 14 ppt salinity were found to have telocytes that shed secretory vesicles, exosomes, and multivesicular bodies into the intraepithelial lymphatic space. Intraepithelial telocytes were also observed to have established planar contact with chloride cells (Fig. 5A–D).

**Figure 6:**
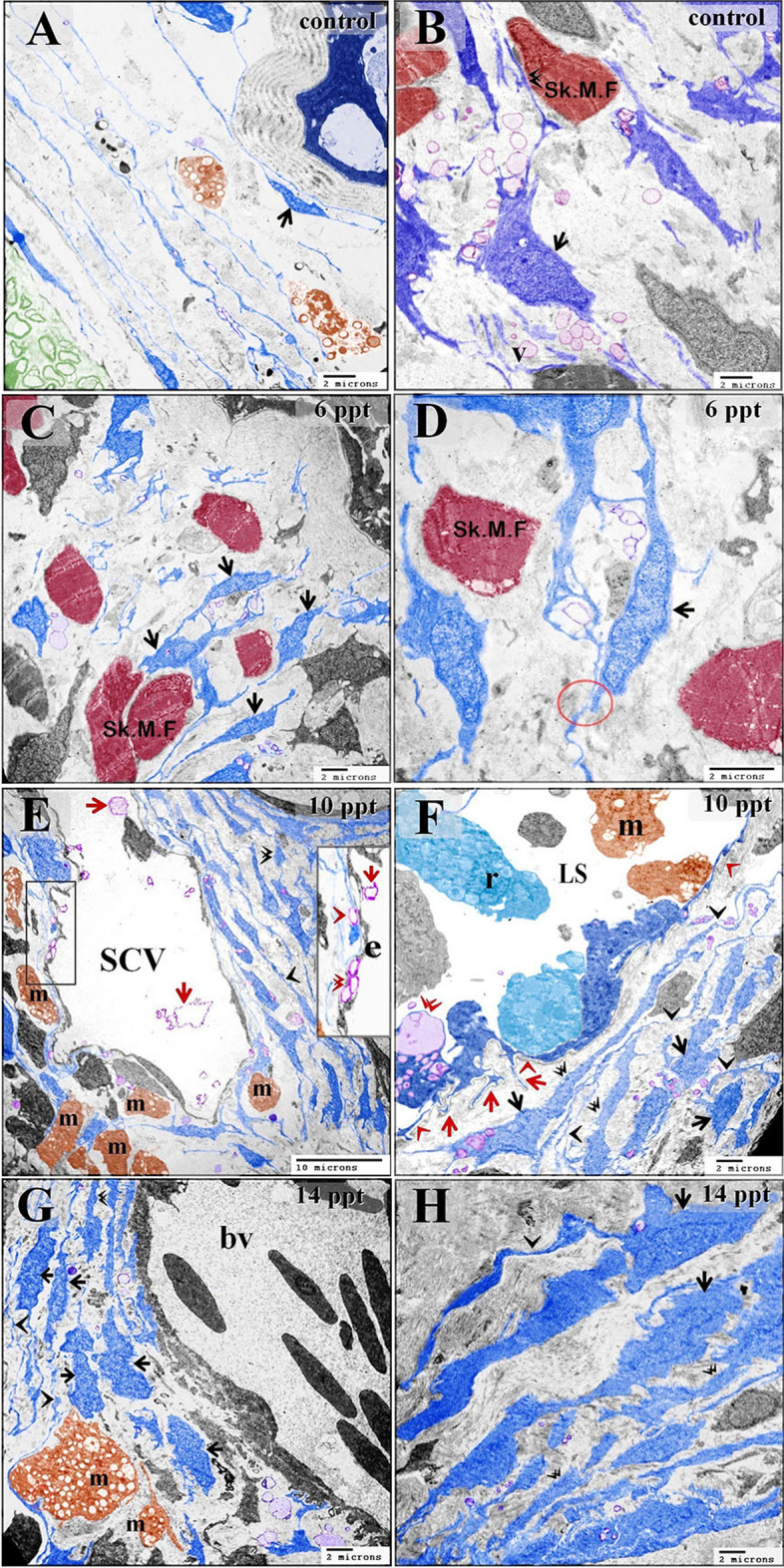
Effect of salinity on stromal telocytes. Colored ultrathin sections prepared from the gill arches of fish exposed to 0.2 ppt (control) (**A**, **B**), 6 ppt (**C**, **D**), 10 ppt (**E**, **F**), and 14 ppt (**G**, **H**) salinity. (**A**, **B**) Telocytes appeared as small, spindle- or satellite-shaped cells (arrows). A telocyte establishing direct contact with skeletal muscle is shown (double arrowhead). (**C**, **D**) Small, spindle-shaped telocytes are shown establishing a homocellular junction (red circle). (**E**) A large population of telocytes is shown surrounding a secondary circulatory vessel (SCV). Telocyte secretory vesicles (red arrowheads) were transported through the endothelial lining of a secondary circulatory vessel (red double arrowheads) and shed into the lumen of the vessel (red arrows). Note the thick telopodes (black double arrowhead), slight waviness (black arrowhead) of the telopodes, and macrophages (m). (**F**) Subepithelial telocytes (black arrows). The telopodes were enormous (black arrowheads) and thickened in certain areas (double arrowheads). Note the basal lamina (red arrowheads), basal intraepithelial telocytes giving rise to short basal telopodes (red arrows), podom of the basal intraepithelial telocyte (double arrowhead), lymphatic space (LS), rodlet cells (r), and macrophages (m). (**G**, **H**) The most prominent features that resulted from exposure to high salinity were cell bodies with irregular surfaces (arrows), wavy telopodes (arrowheads), and thickened telopodes (double arrowhead). Note the enlarged macrophage (m) filled with lysosomes and the blood vessel (bv).

**Figure 7:**
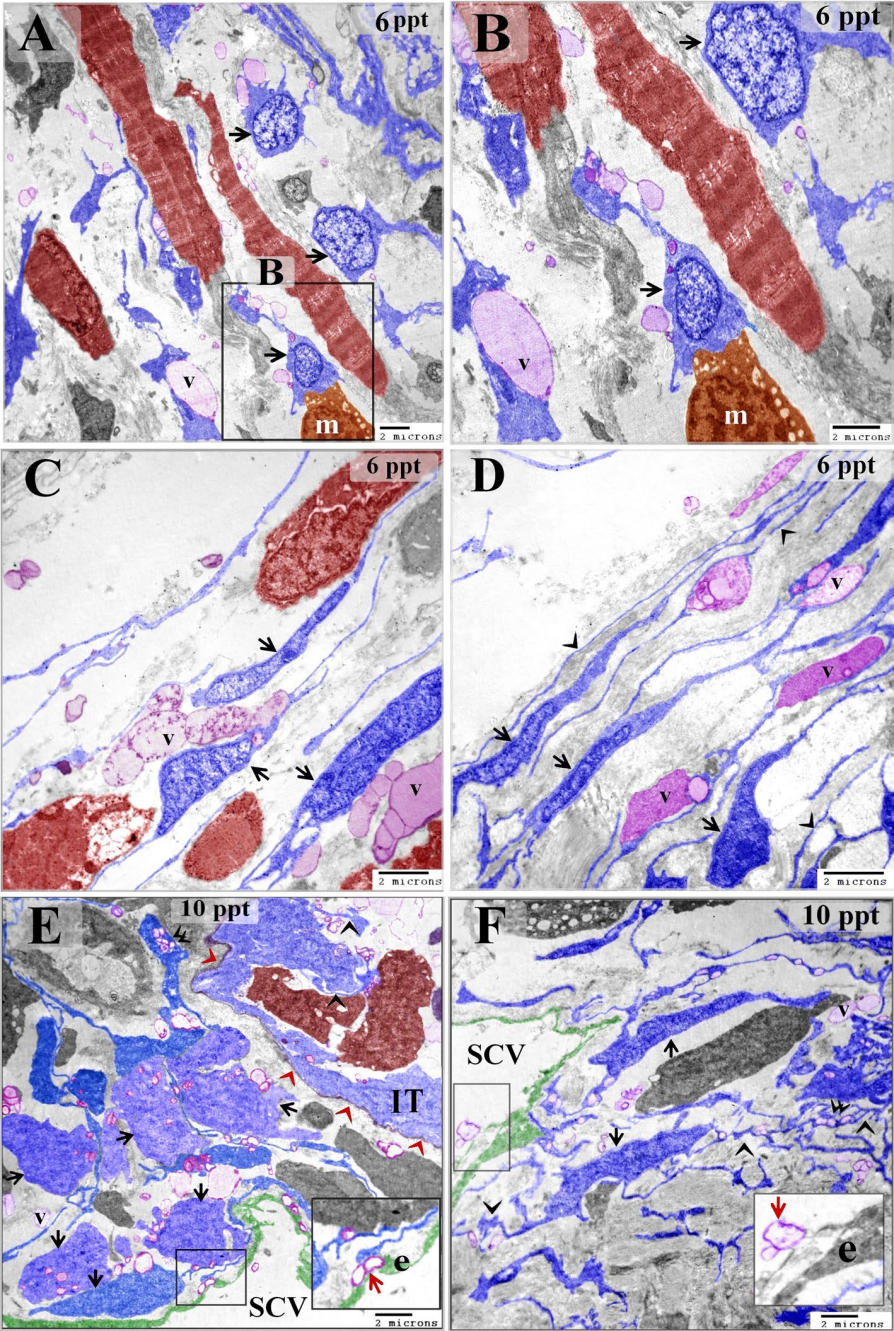
Images showing the increased secretory activity in stromal telocytes responding to increases in salinity and the release of secretory vesicles into secondary circulatory vessels. Colored ultrathin sections prepared from the gill arches of fish exposed to 6 ppt (**A**–**D**) and 10 ppt (**E**, **F**) salinity. (**A**, **B**) Enlarged telocytes that acquired a round or triangular shape (arrows). Note the large secretory vesicle (V) and the cell body of a stromal telocyte establishing contact with a macrophage (m). (**C**, **D**) Spindle-shaped telocytes (arrows) shedding large secretory vesicles (V). Note the enormous telopodes (arrowheads). (**E**) Numerous enlarged subepithelial telocytes (arrows). Some telocytes established contact with the endothelial lining of a secondary circulatory vessel (SCV). Note the podom (double arrow), the transfer of telocyte secretory vesicles (red arrow) to the cytoplasm of endothelial cells (e), the basal lamina (red arrowheads), and the intraepithelial telocytes (IT) and their telopodes (arrowheads). (**F**) Spindle-shaped stromal telocytes (arrows) and their telopodes are shown forming an extensive network (arrows), podom (double arrowhead), and secretory vesicles (V). Note the secretory vesicles (red arrow) in the lumen of the secondary circulatory vessel (SCV) and the endothelial cells (e).

In the control samples, the cell bodies of the stromal telocytes were small and spindle-shaped, satellite-shaped, round, or triangular with thin telopodes. The telopodes consisted of podoms and podomers. The macrophages in these samples were small and contained vesicles (Figs. 6A,B; 7A,B). The telocytes had established homocellular junctions (Fig. 6C,D). As the salinity increased across the experimental groups, increasing morphological modifications were observed in the stromal telocytes. Enlarged cell bodies were observed at 6 ppt salinity (Fig. 3C–H), and 10 ppt salinity (Fig. 4A,F). At 10 ppt salinity, thickened and slightly wavy telopodes were apparent (Fig. 6E,F). The telopodes frequently formed an extensive network (Fig. 7F). The main changes observed to occur with increasing salinity were the presence of wavy and thickened telopodes and telocytes with uneven surfaces (Fig. 6G,H). The stromal telocytes exhibited greater secretory activities when the salinity reached 6, 10, and 14 ppt (Fig. 7A–D). They also established contact with the endothelial lining of the secondary circulatory system or lymphatic vessels, and their secretory vesicles emptied in proximity to these vessels. Trans-endothelial transportation of the secretory vesicles was observed in the secondary circulatory vessels. The transferred vesicles were shed in the lumen of the secondary circulatory pathway (Figs. 3G; 5E; 7E,F).

Both intraepithelial and stromal telocytes had established contact with macrophages via telopodes or the cell body (Figs. 3H; 4A,F). In the control samples, few macrophages were detected in the gill arch stroma (Fig. 6A,B). Macrophages became more active and were rich in lysosomes and vesicles in samples from fish exposed to 6 ppt salinity (Fig. 3F–H). In samples from fish exposed to 10 ppt salinity, there were large numbers of macrophages in the gill arch stroma (Fig. 4A–F) and epithelial lymphatic space (Figs. 4C, 6E,F). In samples from fish exposed to 14 ppt salinity, massive lysosome-rich macrophages were observed in the stroma and epithelial lymphatic space (Figs. 5B, 6G).

**Figure 8:**
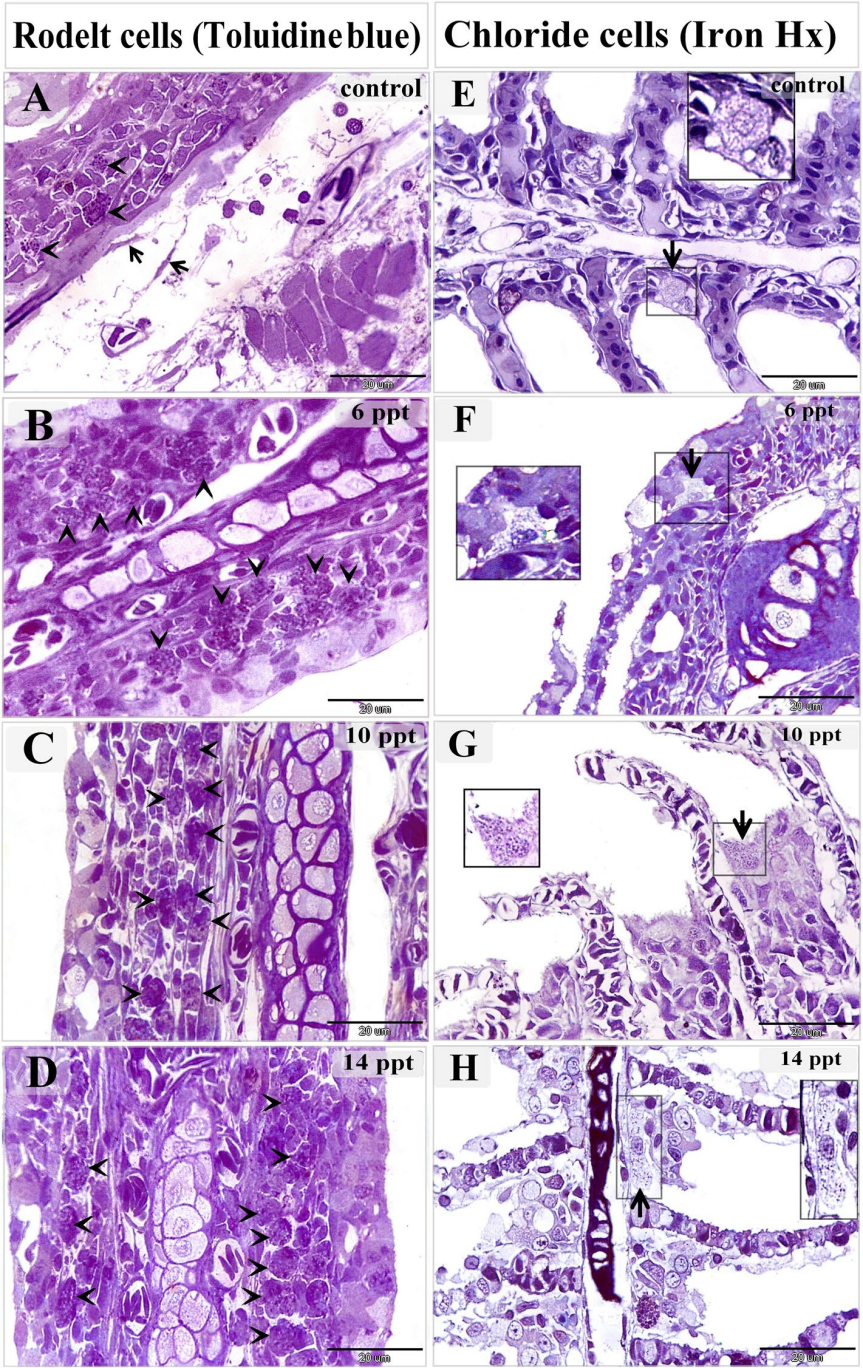
Changes in rodlet and chloride cells responding to salinity stress. Semi-thin sections prepared from gill arches and filaments were stained with toluidine blue (A–D) or Heidenhain’s Iron-Hx (**E**–**H**). (**A**) Immature rodlet cells (granular stage) (arrowheads) were scarce in the control samples. (**B**) The number of immature rodlet cells (arrowheads) increased in the branchial epithelium after exposure to 6 ppt salinity. (**C**) There was a considerable number of rodlet cells (arrowheads) in the branchial epithelium after exposure to 10 ppt salinity. (**D**) There was a significant number of rodlet cells (arrowheads) in the branchial epithelium after exposure to 14 ppt salinity. (**E**) A chloride cell (arrow) in a control sample. Such cells were small and contained few mitochondria, which appeared as dark dots when stained with iron-Hx. (**F**) A large chloride cell (arrow) in a sample from a fish exposed to 6 ppt salinity showing an increased quantity of mitochondria. (**G**) An enlarged chloride cell (arrow) with abundant mitochondria after exposure to 10 ppt salinity. (**H**) A hypertrophic chloride cell (arrow) with considerable mitochondrial content after exposure to 14 ppt salinity.

**Figure 9:**
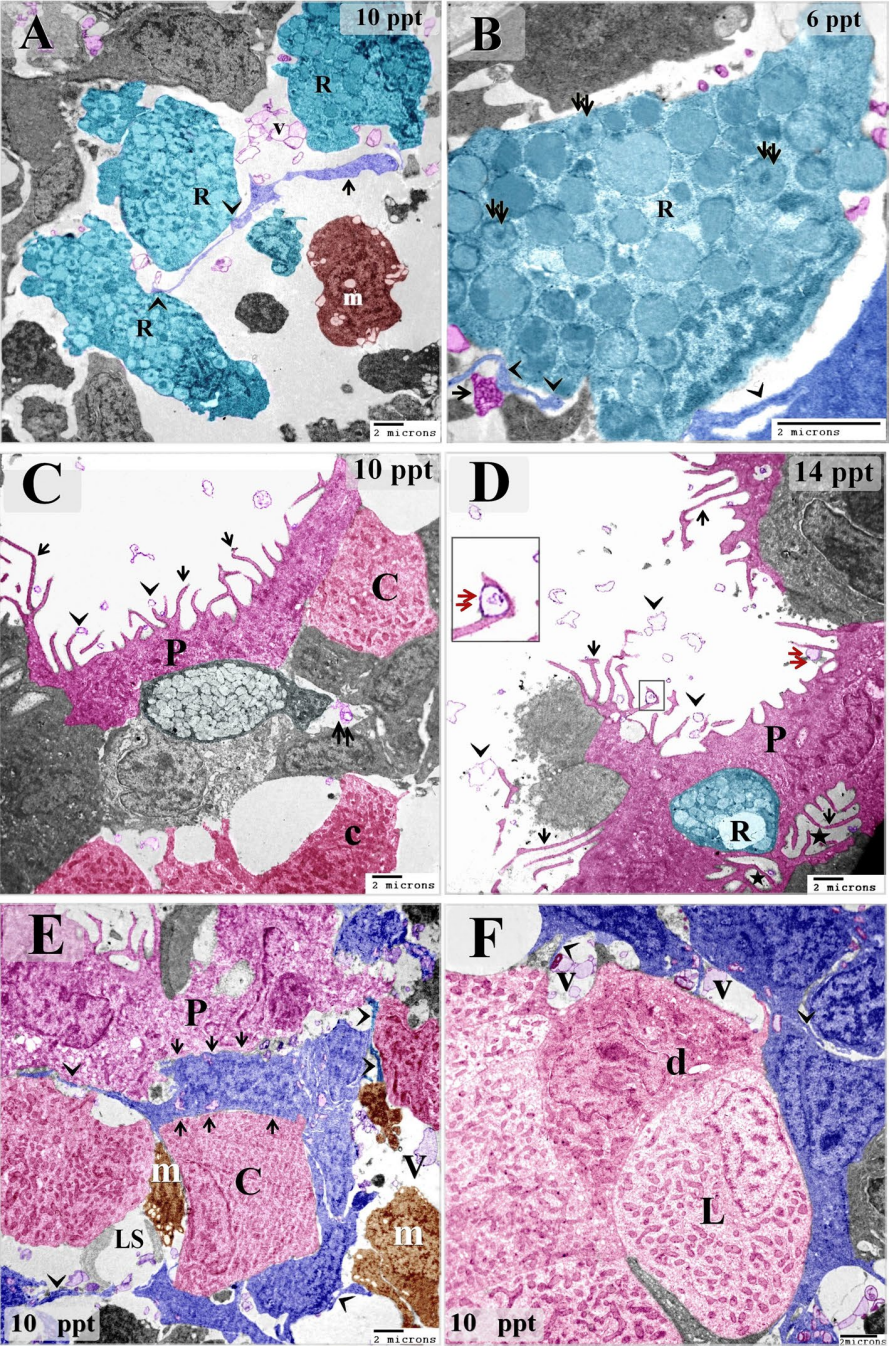
Images illustrating the relationships among intraepithelial telocytes and rodlet, chloride, and pavement cells. Colored ultrathin sections prepared from gill arches of fish exposed to 6 ppt (**B**), 10 ppt (**A**, **C**, **E**, **F**), and 14 ppt (**D**) salinity. (**A**) Stromal telocytes (arrow) in direct contact with rodlet cells (arrowheads). Note the telocytes shedding secretory vesicles (V) in the vicinity of rodlet cells (R). Note the macrophage (m). (**B**) Rodlet cell (granular stage) with immature rodlet granules (double arrows) exhibiting electron-dense central cores. Note the telopodes (arrowheads) in contact with the rodlet cell (R) and the multivesicular body (arrow). (**C**, **D**) Pavement cells (P) from fish exposed to 10 and 14 ppt salinity had long microvilli (arrows), which delivered telocyte secretory vesicles (arrowheads) to the surface of the branchial epithelium. Not the surface invaginations or pits (asterisk) in the pavement cells, the secretory vesicles in the lymphatic space (double arrow), the chloride cells (C), and the rodlet cell (R). (**E**) An intraepithelial telocyte forming planar contacts (arrows) with pavement cells (P) and a chloride cell (C). Note the telopodes (arrows), macrophages (m), secretory vesicles (V), and lymphatic space (LS). (**F**) Two types of mitochondria-rich chloride cells, dark (d) and light (L), are shown connected to telocytes. Note the telopodes (arrows) and secretory vesicles (V).

Intraepithelial and stromal telocytes were found to form contacts with immature rodlet cells (granular rodlet cells) (Figs. 3F–H, 4A–D, 8A–D, 9A,B), and telocytes shed secretory vesicles and multivesicular bodies in the vicinity of immature rodlet cells (Fig. 9A,B). Secretory vesicles from telocytes were observed in the surface epithelium (Fig. 9C,D). In addition, intraepithelial telocytes were observed to establish planar contact with pavement cells (Fig. 9E). In the control samples, the pavement cells were flattened and had short microvilli (Fig. 3A). However, modifications were observed in the pavement cells from the fish that were exposed to increasing salinity. Enlarged pavement cells were present in the samples from fish exposed to 6 ppt salinity (Fig. 9C), cuboid-shaped pavement cells were present in the samples from fish exposed to 10 ppt salinity (Fig. 9E), and elongated and columnar pavement cells were found in samples from fish exposed to 14 ppt salinity (Fig. 5A). The micro-ridges of the pavement cells were thin and elongated, extended beyond the epithelial surface, and attached to or enclosed telocyte secretory vesicles. Furthermore, pit-like invaginations were apparent on the surfaces of pavement cells (Fig. 5C,D).

**Figure 10:**
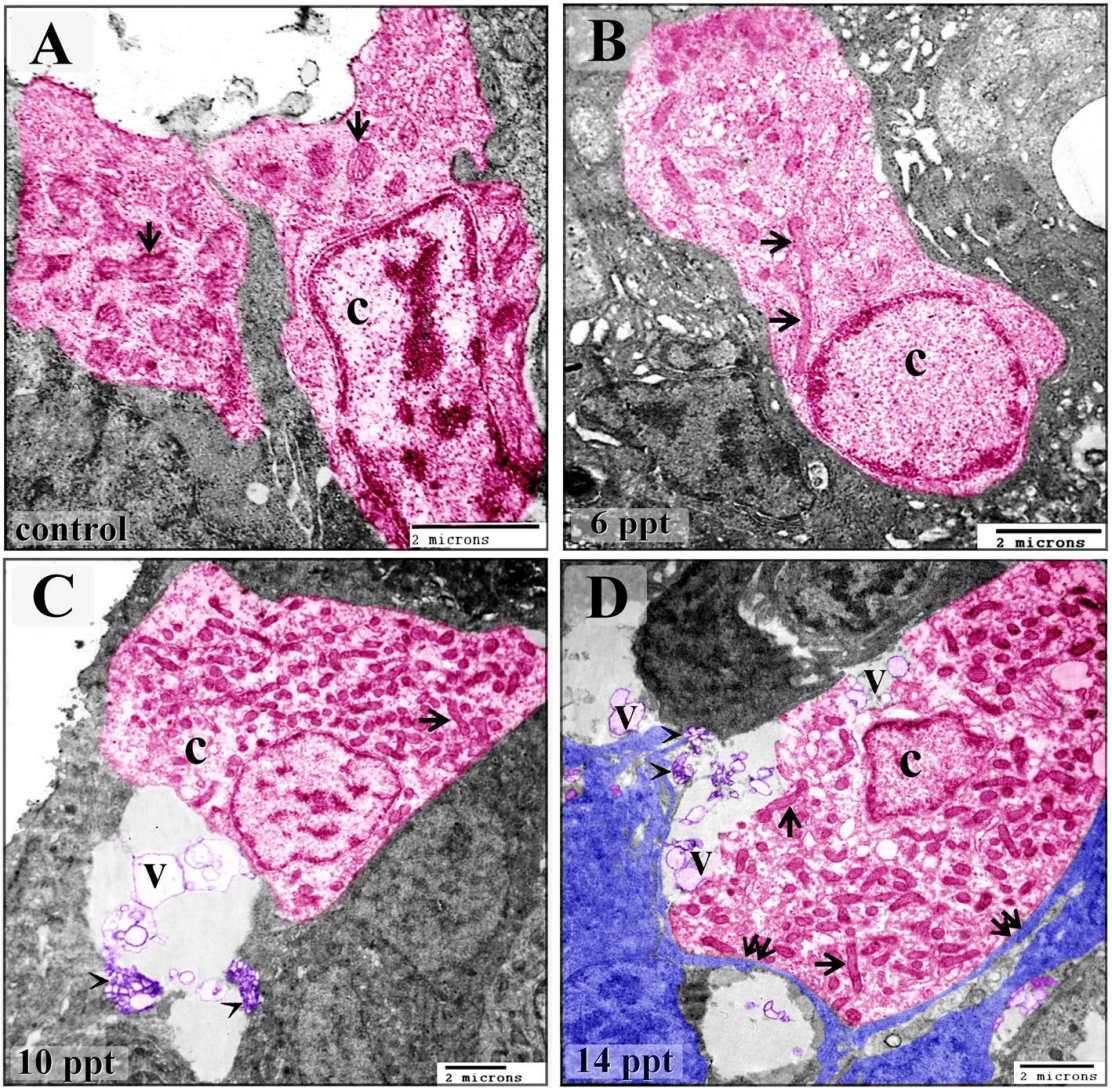
Changes in chloride cells in response to salinity stress. Colored ultrathin sections prepared from the gill arches of fish exposed to 0.2 ppt (control) (**A**), 6 ppt (**B**), 10 ppt (**C**), and 14 ppt (**D**) salinity. (**A**) An elongated chloride cell (C) containing relatively few oval-shaped mitochondria (arrows). (**B**) An enlarged and elongated chloride cell (C) exhibiting morphological changes, namely, an increase in the number of mitochondria and elongated and cigar-shaped mitochondria (arrows). (**D**) An enlarged, cuboid chloride cell (C) with numerous mitochondria, some of which were elongated and cigar-shaped (arrows). Note the telocyte secretory vesicles in the vicinity of the chloride cell. (**E**) A hypertrophic, oval-shaped chloride cell (C) with a massive mitochondrial content. Some mitochondria were cigar-shaped (arrows). Note that the telocytes were close to the chloride cells. Note the telopodes (double arrows) and secretory vesicles (V).

Intraepithelial telocytes established planar contacts with chloride cells (Figs. 4D, 5C, 9E,F). As the salinity increased, the chloride cells were observed to undergo structural modifications. TEM imaging showed that they became enlarged and increased in number gradually as the salinity increased. Changes in the mitochondria were also noted when the treated and control samples were compared. There were greater numbers of mitochondria in the treated samples, and the treated samples contained elongated, cigar-shaped mitochondria rather than round or oval-shaped mitochondria, as were found in the control samples. Chloride cells delivered the secretory vesicles of the telocytes, which were transferred through the intraepithelial lymphatic space (Figs. 4C, 5A, 10A–D). The mitochondrial content of the chloride cells was also evaluated using Heidenhain’s Iron-Hx stain. Mitochondria appeared as black granules and increased with the salinity level (Fig. 8E–H).

## Discussion

This study was conducted to evaluate the telocyte response to salinity stress and its impact on osmoregulatory and immune cells. We detected telocytes in semi-thin sections using toluidine blue, methylene blue, and Heidenhain’s Iron-Hx staining, and we used ultrathin sections to examine ultrastructural modifications in telocytes in relation to epithelial and stromal cells.

It was found that the examined telocytes underwent morphological changes during salinity stress. The telocytes in the control samples were spindle-shaped and had fine telopodes. No significant changes were observed in telocytes from fish exposed to 6 ppt salinity; however, some telocytes exhibited increased secretory activity. Telocytes from fish exposed to 10 and 14 ppt salinity were observed to shed large secretory vesicles and have an enlarged cell body and thicker telopodes that formed extensive networks in some cases. Hormones may affect the secretory activities of telocytes, and increased telocyte secretory activity has been reported in melatonin-treated ram seminal vesicles^23^.

In this study, we detected two types of telocytes in the gills of the common carp—intraepithelial and stromal telocytes—and these were found in specific locations. Their telopodes mediated homocellular and heterocellular contacts, and heterocellular contacts were formed with a wide range of cells and structures.

Intraepithelial telocytes were found to form labyrinthine networks and the boundaries of the lymphatic space, which is comprised of interconnected channels interspersed among epithelial cells. The intraepithelial telocytes were observed to shed their secretory vesicles and multivesicular bodies into the intraepithelial lymphatic space, which in turn delivered them to other epithelial and immune cells, including chloride, pavement, mucous, and rodlet cells, as well as macrophages. It appears that intraepithelial telocytes may also establish contact with epithelial and immune cells via point or planar contacts. Intraepithelial telocytes were previously detected by scanning electron microscopy in the bovine uterine tube. Telocytes have been found in the basal layer of epithelial cells, with telopodes that extended among the epithelial cells^24^.

Our results show that telocytes establish intercellular communication with chloride cells by either direct contact or a paracrine mechanism and thus reveal that telocytes may play a role in osmoregulation. The salinity of the water affected the morphology of the telocytes, which in turn influenced the chloride cells. We have shown that they undergo hypertrophy and that their mitochondria undergo morphological changes and increase in number as salinity increases. Similar results have been documented in the Hawaiian goby (*Stenogobius hawaiiensis*): salinity caused a slight increase in chloride cell number and size in that species^25^. Gill chloride cells regulate ionic transportation via transport proteins, which have a polarized distribution. Three types of transport proteins have been identified in chloride cells: CFTR (an anion channel), NKCC, and Na^+^/K^+^-ATPase (a sodium–potassium pump). These three proteins were also found to be expressed in gill chloride cells of *S*. *hawaiiensis* acclimated to freshwater and 20 ppt and 30 ppt salinity for 10 days; Na^+^/K^+^-ATPase and NKCC had a basolateral/tubular localization, and CFTR was expressed at the apical surface^25^. The authors of that study concluded that Na^+^/K^+^-ATPase expression is not affected by salinity and that CFTR immunoreactivity increases with salinity.

In the present study, pavement cells were also found to be connected to telocytes. Telocyte secretory vesicles were observed to reach the surface epithelium and attach to or be partially enclosed by the micro-ridges of the pavement cells. These cells were flattened in the control samples and gradually enlarged as the salinity increased until they became columnar in shape when the salinity reached 14 ppt. The micro-ridges of the pavement cells became thinner and were elongated at 10 ppt and 14 ppt salinity. The pavement cells also developed surface invaginations at 14 ppt salinity. A similar result was reported in a study conducted with gill epithelia from the Adriatic sturgeon, *Acipenser naccarii*: pavement cells acquired a complex system of micro-ridges on their apical surface during exposure to a hypertonic environment (salinity 35 ppt)^26^. Pavement cells play an important role in gas exchange^27^ and are rich in proton pumps, which regulate acid–base balance^21,22^.

Regarding the common carp’s ability to survive in highly saline water, in our study, this species had difficulty surviving when the salinity was more than 10 ppt. A high mortality rate was observed at 12 ppt salinity, and there was a marked increase in the mortality rate at 14 ppt salinity. Mangat and Hundal investigated the effect of salinity on* C*. *carpio* survival in different seasons. They exposed fish to 0, 1.5, 3, 6, or 12 ppt salinity for 60 days. All the fish were viable and survived at 0 ppt to 6 ppt salinity during all seasons. The mortality rate was 50% at 12 ppt salinity during winter (14.50–19.00 °C) and reached 100% during summer (28.00–37.00 °C) and autumn (22.50–30.50 °C)^28^.

We also examined the relationships that intraepithelial and stromal telocytes had with immune cells. Our results showed that the telocytes maintained relationships with immune cells, particularly macrophages and rodlet cells, via cellular contact and paracrine signaling. This indicates that telocytes may maintain and enhance the immune response at different salinity levels. Samples from fish exposed to 14 ppt salinity were found to contain more and larger macrophages that had increased phagocytic activity and were rich in lysosomes and vesicles. The connection between telocytes and macrophages has previously been documented in studies on the mouse eye and rat urinary tract^29,30^. In another study that was conducted using human heart tissue, the contact point between these two cell types was identified as an electron-dense nanostructure^12^. Furthermore, mouse peritoneal macrophages were shown to be activated and secrete cytokines and enzymes when co-cultured with telocytes in conditioned media^31^. Macrophage activity has been investigated in relation to salinity level and ration. The phagocytic activity of macrophages from black sea bream, *Mylio macrocephalus* (Basilewsky), juveniles is primarily affected by ration size rather than salinity^32^.

Our results also indicate that intraepithelial and stromal telocytes interact with immature rodlet cells (granular stage). Telocytes exert their effect on rodlet cells through either direct contact or paracrine signaling. Hence, when telocytes undergo modifications in response to high salinity, rodlet cells may also be affected. Indeed, increases in the number of rodlet cells were noted in samples from fish exposed to 6, 10, and 14 ppt salinity. Thus, our findings suggest that telocytes may have a role in the regulation of biological activities and the maturation of rodlet cells. Many studies have investigated the nature and function of rodlet cells. Rodlet cells are thought to act as ion transporting cells and be involved in osmoregulation^33^, and it is widely accepted that rodlet cells participate in the immune response. They are present when a response is mounted against helminthic infestations and other noxious agents and are considered a type of eosinophilic granulocyte^34,35^. Rodlet cells have been shown to undergo significant salinity-dependent changes; for example, rodlet cells reportedly increase in number in European sea bass, *Dicentrarchus labrax*, when salinity decreases^36^.

In this study, stromal telocytes were observed to establish direct contact with secondary vascular vessels or lymphatic vessels. Secretory vesicles from stromal telocytes were observed in lymphatic vessels. Lymphatic vessels deliver the inflow from arterial vessels via arterio-arterial anastomoses and drain into the venous circulation^37^. Thus, our findings suggest that the secondary vascular vessels act as the principal pathway for the delivery of telocyte secretions to the blood and that telocytes may exert effects on remote tissues and organs via a paracrine mechanism. In addition, the secondary vascular vessels are implicated in gaseous exchange and ion transport^38^.

## Conclusions

In conclusion, fish may accommodate salinity changes by activating an adaptive response via cell-to-cell communication. Telocytes are a major component of this communication system, as they regulate the function of a wide variety of cells via direct contact and paracrine signaling. In this study, as the salinity increased, the telocytes underwent morphological modifications and increased their activities that influence epithelial, immune, and stromal effector cells. Intraepithelial telocytes were shown to affect chloride cells, pavement cells, immature rodlet cells, and macrophages, while stromal telocytes influenced stem cells, skeletal myoblasts, macrophages, and rodlet cells. Thus, telocytes enhanced immunity, osmoregulation in the gill lamellar and filament epithelium, and regeneration of stromal cells. As a result, the fish could survive and maintain internal homeostasis in hyperosmotic environments up to 10 ppt salinity.

## Materials and methods

The protocols of this study were approved by the National Ethics Committee of South Valley University and veterinary authorities in South Valley University Province, Egypt (Approval NO. 8a/13.12.2020).

### Fish source and transportation

The fish (common carp, *Cyprinus carpio*) were obtained from a private fish farm at El-Dakahlea Government and transported in large water tanks. During transportation, the oxygen level was maintained at 5 mg/L, the water tank temperature was 23 ± 3 °C, and the pH of the water was 7.2–7.5.

### Fish acclimation

Apparently healthy one-month old fingerlings 7 ± 2 cm in length and with a body weight of 10 ± 2 g were used. The fish were collected and transported to the wet laboratory at the Faculty of Veterinary Medicine, South Valley University, Qena, Egypt. The fish were maintained under laboratory conditions during the adaptation period, which ran for three weeks before the experiment was conducted. The fish were maintained in running water (salinity = 0.2 parts per thousand [ppt]) and fed twice daily (to ad libitum) with a commercial floating powdered feed containing 45% protein and a feeding rate of 3% of their body weight.

### Aquaria

The fish were originally kept in a re-circulating system in porcelain aquaria (260 × 65 × 70 cm) according to the protocol for maintaining bioassay fish, as was previously described (Ellsaesser and Clem, 1986)^39^. Experiments were conducted in fiberglass aquaria (60 × 30 × 40 cm). The dissolved oxygen level was maintained above 5 mg/L, the water temperature was kept at 23 ± 3 °C, and the water pH value was 7.2–7.5.

### Salinity exposure

A total of 36 acclimated, apparently healthy common carp with a body weight range of 9–11 g were selected for the experimental groups. The fish were divided into four experimental groups, each containing nine fish. Three fish were placed in each of 12 fiberglass aquaria (60 × 30 × 40 cm), creating three replicates for each group. Three groups were gradually subjected to three different salinities, reaching 6, 10, or 14 ppt, with a 2 g/L NaCl increase every two days. The fourth group was reared in freshwater (dechlorinated tap water with 0.2 ppt salinity level) and used as the control group. The aquarium water was replaced every two days with water that had the desired salinity, and the aquaria were cleaned at the same time. Salinity was checked and adjusted regularly (every two days) in between the water changes. When the desired salinity levels were reached, the fish were left to acclimate to the new salinities for a minimum of two weeks before sample collection.

### Clinical examination of fish

The fish were observed daily during the course of the experiment for any apparent clinical signs, lesions, or mortality. The mortality rate was calculated from the number of dead fish found between each sampling period.

### Fish sampling

At the end of the experimental period, the nine fish in each group were decapitated. Gill filaments and gill arches from both sides were dissected and fixed in glutaraldehyde (10 mL 2.5% glutaraldehyde and 90 mL 0.1 M Na-phosphate buffered formalin).

### Preparation of resin-embedded specimens for semi-thin and ultrathin sectioning

The fixed gill filament and arch samples were cut into small pieces. They were washed four times for 15 min in 0.1 M sodium phosphate buffer (pH 7.2) and then post-fixed in 1% osmic acid in 0.1 M Na-phosphate buffer at 4 °C for 2 h. The osmicated samples were washed three times for 20 min in 0.1 M phosphate buffer (pH 7.2). Dehydration was performed using graded aceton (70, 80, 90, 100%), with samples exposed to each concentration for 10 min. The dehydrated samples were immersed in a mixture of aceton/resin (1/1 for one day, 1/2 for another day) and in pure resin for three days. The resin was prepared using 10 g ERL, 6 g diglycidyl ether of polypropyleneglycol (DER), 26 g nonenyl succinic anhydride (NSA), and 0.3 g dimethylaminoethanol (DMAE), and thorough mixing using a shaker. The specimens were embedded in the resin at 60 °C for three days. The polymerized samples were cut into semi-thin sections using an ultramicrotome (Ultracut E, Reichert-Leica, Germany) and stained with toluidine blue^40^.

The semi-thin sections were used in histochemical studies. The sections were treated with a saturated alcohol solution of sodium hydroxide for 15 min to dissolve the resin^41^. The semi-thin sections were stained with Heidenhain’s Iron-Hx^42^, and methylene blue was used to stain paraffin sections and prepared as a stain for the semi-thin sections^40^.

Ultrathin sections were obtained using a Reichert ultramicrotome. The sections (70 nm) were stained with uranyle acetate and lead citrate and examined by transmission electron microscopy (TEM; JEOL100CX II) at the central laboratory unit of South Valley University.

### Coloring of images

TEM images were colored using a photo filter 6.3.2 program. The coloring of the images required the color balance to be changed and the use of the stamp tool to color the objective cells. This is a common method used by researchers to add color to images^43–47^.

The study was conducted in compliance with the Animals in Research: Reporting In Vivo Experiments (ARRIVE) guidelines^48^. All methods were performed in accordance with the relevant guidelines and regulations by.

## Ethics approval

The protocols of this study were approved by the National Ethics Committee of South Valley University and veterinary authorities in South Valley University Province, Egypt (Approval No. 8a/13.12.2020).

## Data Availability

The data sets collected and/or analyzed during the current study are available from the corresponding authors on reasonable request.

## Abbreviations

CFTR: Cystic fibrosis transmembrane conductance regulator
DER: Diglycidyl ether of polypropyleneglycol
DMAE: Dimethylaminoethanol
ECC: 3,4-Epoxycyclohexylmethyl-3′,4′-epoxycyclohexane carboxylate, also known as 3,4 and cycloaliphatic epoxide resin (ERL 4221)
NaCl: Sodium chloride
NKCC: Na–K–2Cl cotransporter
NSA: Nonenyl succinic anhydride
ppt: Parts per thousand
st: Stratum compactum
V: Secretory vesicles
P: Pavement cells
SCV: Secondary circulatory vessels
RBC: Red blood cells
r: Rodlet cells
m: Macrophages
bc: Blood capillaries
LS: Lymphatic space
mu: Mucous cell
C: Chloride cell
